# Adipose-targeted SWELL1 deletion exacerbates obesity- and age-related nonalcoholic fatty liver disease

**DOI:** 10.1172/jci.insight.154940

**Published:** 2023-03-08

**Authors:** Susheel K. Gunasekar, John Heebink, Danielle H. Carpenter, Ashutosh Kumar, Litao Xie, Haixia Zhang, Joel D. Schilling, Rajan Sah

**Affiliations:** 1 Department of Internal Medicine, Cardiovascular Division, Washington University School of Medicine, St. Louis, Missouri, USA.; 2 Department of Pathology, Saint Louis University School of Medicine, St. Louis, Missouri, USA.; 3 John Cochran VA Medical Center, St. Louis, Missouri, USA.

**Keywords:** Hepatology, Metabolism, Adipose tissue, Glucose metabolism, Liver cancer

## Abstract

Healthy expansion of adipose tissue is critical for the maintenance of metabolic health, providing an optimized reservoir for energy storage in the form of triacylglycerol-rich lipoproteins. Dysfunctional adipocytes that are unable to efficiently store lipid can result in lipodystrophy and contribute to nonalcoholic fatty liver disease (NAFLD) and metabolic syndrome. Leucine-rich repeat containing protein 8a/SWELL1 functionally encodes the volume-regulated anion channel complex in adipocytes, is induced in early obesity, and is required for normal adipocyte expansion during high-fat feeding. Adipose-specific SWELL1 ablation (Adipo KO) leads to insulin resistance and hyperglycemia during caloric excess, both of which are associated with NAFLD. Here, we show that Adipo-KO mice exhibited impaired adipose depot expansion and excess lipolysis when raised on a variety of high-fat diets, resulting in increased diacylglycerides and hepatic steatosis, thereby driving liver injury. Liver lipidomic analysis revealed increases in oleic acid–containing hepatic triacylglycerides and injurious hepatic diacylglyceride species, with reductions in hepatocyte-protective phospholipids and antiinflammatory free fatty acids. Aged Adipo-KO mice developed hepatic steatosis on a regular chow diet, and Adipo-KO male mice developed spontaneous, aggressive hepatocellular carcinomas (HCCs). These data highlight the importance of adipocyte SWELL1 for healthy adipocyte expansion to protect against NAFLD and HCC in the setting of overnutrition and with aging.

## Graphical abstract

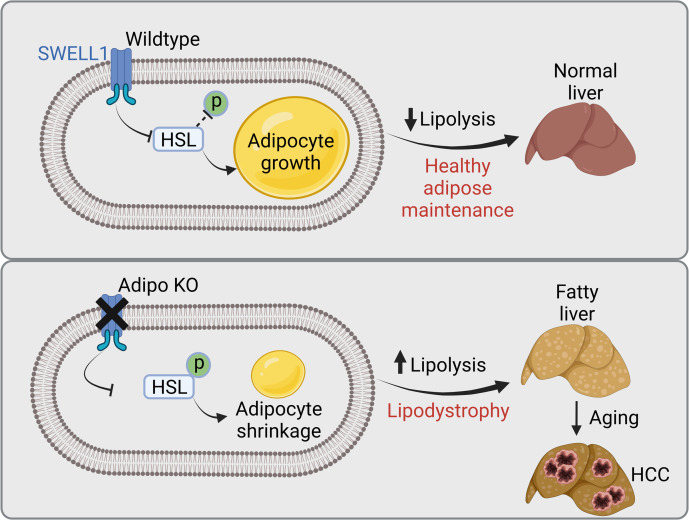

## Introduction

Nonalcoholic steatohepatitis (NASH) is a significant global health concern affecting 41 million adults in the United States and European Union alone (1). NASH is associated with hepatic fibrosis, cirrhosis, and hepatocellular carcinoma (HCC) and is currently the second most common indication for liver transplantation in the United States, after hepatitis C (1, 2), with direct costs of care estimated to be more than $16 billion per year in the United States and European Union (3–5). Type 2 diabetes (T2D), which is characterized by insulin resistance of target tissues (fat, skeletal muscle, liver) and, ultimately, impaired insulin secretion from the pancreatic β cell (6–8), is strongly associated with NASH: 44% of patients with NASH have T2D, and 37% of patients with T2D have NASH (9–11). Also, patients with T2D have a significantly more aggressive form of nonalcoholic fatty liver disease (NAFLD) compared with those without T2D, with a 2-fold higher rate of NASH, cirrhosis, and death and an 8-fold increased rate of HCC (12). T2D itself is reaching epidemic proportions, with more than 29 million with T2D and about 86 million with prediabetes in the United States alone (in 2014, per the CDC) (13). NASH and T2D are also both associated with morbidity and mortality from cardiovascular disease (CVD). CVD is the most common cause of death in patients with NASH (4) and a major cause of death in T2D (14).

NAFLD and its eventual progression to NASH and end-stage HCC is primed by the development of hepatic steatosis. Hepatic steatosis occurs via a number of mechanisms, including hepatic free fatty acid uptake during lipolysis, hepatic de novo lipogenesis, impaired fatty acid oxidation, and diminished lipid disposal (15). Increased lipolysis in insulin-resistant adipose tissue in the setting of obesity further exacerbates hepatic steatosis and highlights the importance of maintaining adipose insulin sensitivity and healthy adipocyte expansion in the setting of overnutrition (16–18). During feeding, calories are stored in the form of triacylglycerol-rich lipoproteins via activation of insulin-regulated lipogenesis pathways. Conversely, during fasting, lipolysis is triggered by stimulation of lipolytic pathways to provide a source of free fatty acids as an energy supply. Therefore, molecular mechanisms that regulate adipocyte insulin sensitivity during adipocyte expansion in the setting of overnutrition are also anticipated to influence fatty acid flux to the liver and hepatic steatosis. Among these molecular mechanisms, it is being increasingly recognized that mechanical forces may stimulate lipogenesis and adipogenesis pathways in cultured adipocytes (19–22), and in primary human adipocytes (23), contributing to a feed-forward amplifier to promote lipogenesis (19, 24). Indeed, a number of mechanoresponsive and mechanosensitive ion channels expressed in adipocytes, including transient receptor potential vanilloid type 4 (TRPV4) and mechanosensitive ion channel Piezo1, have been shown to regulate adipogenesis, adipocyte expansion, and thermogenesis in vitro and in vivo and can influence NAFLD (25–27).

We recently identified SWELL1/leucine-rich repeat containing protein 8a (LRRC8a), as a required component of the putative mechanoresponsive volume-regulated anion channel (VRAC) signaling complex in adipocytes. We hypothesize that the SWELL1/LRRC8 complex senses adipocyte hypertrophy in the setting of overnutrition and potentiates the insulin/PI3K/AKT2 signaling axis in a feed-forward amplifier manner (24). Consequently, adipose-specific SWELL1 ablation (Adipo KO) results in decreased adiposity, exacerbated insulin resistance, and impaired glucose uptake in the setting of obesity (28). In this study, we demonstrate that SWELL1 is required to prevent excessive lipolysis, resultant increases in plasma free fatty acids and glycerol, and accumulation of diacylglycerides and toxic lipid species in the liver that drive the development of NAFLD in mice in the setting of overnutrition. Adipocyte-targeted SWELL1 depletion also predisposes to NAFLD with aging, as well as to spontaneous HCC in males raised on a regular diet. These data reveal a potentially novel adipose SWELL1/liver axis that regulates adipo-hepatic lipid flux that when dysregulated results in NAFLD and HCC in mice.

## Results

### Adipose SWELL1 deletion limits adipose depot expansion with overnutrition.

**Figure 1:**
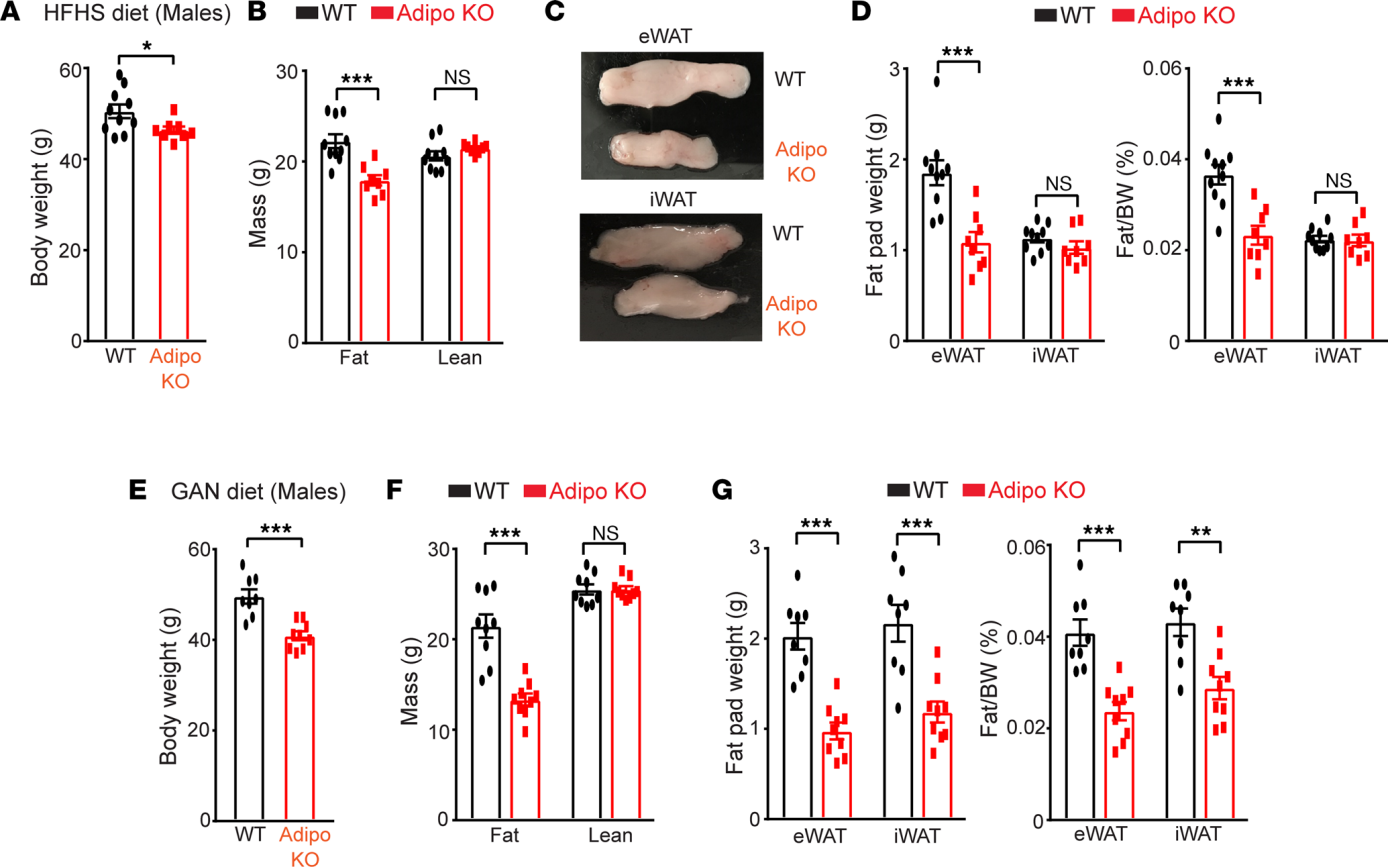
Adipose SWELL1 supports adipose tissue expansion and maintenance. (**A**) Total body weight of WT (*n* = 10) and Adipo KO (*n* = 8) mice (males) fed with high-fat/high-sucrose (HFHS) diet for 27 weeks. (**B**) Body composition for total fat and lean mass estimated by NMR from mice in **A** at 21-week time point of HFHS diet. (**C**) Representative images of epididymal (eWAT) and inguinal (iWAT) fat pads isolated from mice in **A**. (**D**) Total fat pad weights and ratio of fat pad over body weight of WT and Adipo-KO mice from **A**. (**E**) Total body weight of WT (*n* = 8) and Adipo-KO (*n* = 9) mice (males) fed with Gubra Amylin NASH (GAN) diet for 23–25 weeks. (**F**) Body composition for total fat and lean mass estimated by EchoMRI from mice in **E** at 22-week time point of GAN diet. (**G**) Total fat pad weights and ratio of fat pad over body weight of WT and Adipo-KO mice from **E**. Data are represented as mean ± SEM. Two-tailed unpaired *t* test was used in **A**, **B**, and **D**–**G** where *, **, and *** represent *P* < 0.05, *P* < 0.01, and *P* < 0.001, respectively.

We showed previously that mice with adipose SWELL1-specific deletion (Adiponectin-Cre *SWELL1^fl/fl^*; Adipo KO) are indistinguishable with respect to adiposity compared to the wild-type (WT; *SWELL1^fl/fl^*) controls when raised on a regular chow diet (18% kcal fat; 59% kcal carbohydrate) (28, 29) for 12 weeks. However, when raised on a high-fat diet (HFD; 60% kcal fat), Adipo-KO mice develop significantly less adiposity based on body composition and adipose tissue mass, driven by reduction in adipocyte size (28). In this study, we raised WT control and Adipo-KO mice on either high-fat, high-sucrose (HFHS, 58% kcal fat, 18% sucrose) diet or Gubra Amylin NASH (GAN, 40% kcal fat, 40% kcal carbohydrate) diet to more closely mimic diet-induced NAFLD/NASH models (30, 31). Similar to our previous findings in regular HFD-fed mice (28), Adipo-KO mice raised on HFHS diet for 27 weeks gained less weight than WT mice (Figure 1A), which was driven by an approximately 20% reduction in total fat mass (Figure 1B), with no change in lean mass as assessed by NMR (Figure 1B). This reduction in fat mass is largely attributed to a 42% decrease in epididymal white adipose tissue (eWAT) fat pad weights while the inguinal white adipose tissue (iWAT) fat content remained unchanged (Figure 1, C and D). Adipo-KO mice raised on a GAN diet for 23–25 weeks also gained less weight (Figure 1E), driven by a 38% reduction in total fat mass (Figure 1F), with no change in lean mass (Figure 1F), as assessed by EchoMRI. In contrast to HFHS diet, this reduction in fat mass in GAN-fed Adipo-KO mice was associated with a 52% and 45% reduction in both eWAT and iWAT depots, respectively (Figure 1G), compared with controls. The disparities in adipose depot size in mice raised on different diets may be attributed to dietary composition (fat, carbohydrates), as this is known to induce obesity at variable rates (32, 33).

### Adipose SWELL1 ablation augments lipolysis via hormone sensitive lipase activation.

**Figure 2:**
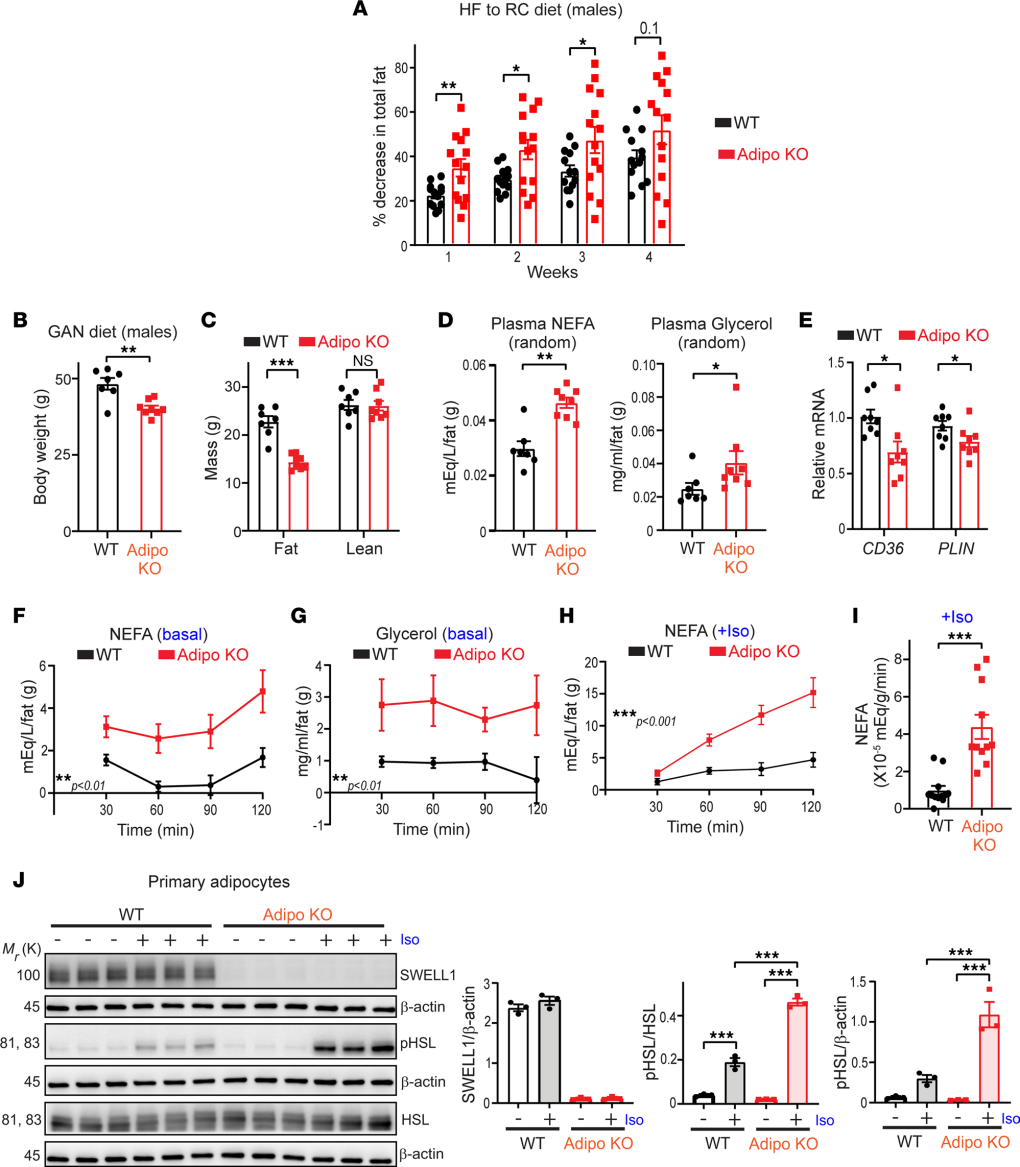
Adipose SWELL1 ablation augments lipolysis and hormone sensitive lipase activation. (**A**) Percentage decrease in total fat in high-fat (HF) diet (6–9 months) WT and Adipo-KO mice (males) after switching to regular chow (RC) diet for 4 weeks estimated by NMR. (**B**) Total body weight of WT (*n* = 7) and Adipo-KO (*n* = 8) mice (males) fed with GAN diet for 22 weeks. (**C**) Body composition for total fat and lean mass estimated by EchoMRI from mice in **B** at 22-week time point of GAN diet. (**D**) Random plasma NEFAs and glycerol normalized to total fat mass of WT and Adipo-KO mice in **B**. (**E**) mRNA expression of *CD36* and *PLIN* relative to *GAPDH* in eWAT isolated from WT and Adipo-KO (*n* = 8 each, males) mice on GAN diet for 22–25 weeks. (**F** and **G**) Ex vivo lipolysis assay measuring NEFA (**F**) and glycerol (**G**) released from eWAT of WT (*n* = 9 experimental replicates from 3 mice) and Adipo-KO mice (*n* = 8 experimental replicates from 3 mice) at 30, 60, 90, and 120 minutes under basal, unstimulated conditions. (**H** and **I**) Ex vivo lipolysis assay measuring NEFA (**H**) released from WT and Adipo-KO (*n* = 11–12 experimental replicates from 2 mice/group) mice on HFD for 5 weeks at 30, 60, 90, and 120 minutes upon stimulation with 50 nM isoproterenol and the corresponding rate of NEFA production (**I**). (**J**) Western blots for protein levels of SWELL1, p-HSL, total HSL, and β-actin from primary adipocytes isolated from *SWELL1^fl/fl^* mice transduced with either Ad-GFP or Ad-Cre-GFP to generate WT and SWELL1-KO primary adipocytes and treated with either vehicle or 100 nM isoproterenol for 15 minutes and the corresponding densitometry analysis. Data are represented as mean ± SEM. Two-tailed unpaired *t* test was used in **A**–**E** and **I**. Two-way ANOVA was used in **F**–**H**. One-way ANOVA was used in **J**. *, **, and *** represent *P* < 0.05, *P* < 0.01, and *P* < 0.001, respectively.

Our previous studies revealed that impaired adipocyte growth observed in Adipo-KO mice is associated with disrupted insulin/PI3K/AKT2 signaling (28). Impaired insulin/PI3K/AKT2 signaling is predicted to not only reduce lipogenesis, but also increase lipolysis, and adipocyte contraction in Adipo-KO mice, under conditions of reduced caloric intake. To directly test this, we performed a weight loss experiment where obese mice raised on HFD for 6–9 months were switched to regular chow (RC) diet for 4 weeks, and body composition was measured by NMR each week to noninvasively track reductions in fat mass. During the first 3 weeks, Adipo-KO mice exhibited a larger proportionate decrease in adiposity than WT mice (Figure 2A), consistent with increased adipose depot contraction, and increased lipolysis. To assess the extent of lipolysis, we measured plasma nonesterified free fatty acids (NEFAs) and glycerol in WT and Adipo-KO mice raised on a GAN diet for approximately 22 weeks. Despite the fact that Adipo-KO mice developed less adiposity (Figure 2, B and C), the absolute values of NEFAs and glycerol were similar to WT mice (Supplemental Figure 1, A and B; supplemental material available online with this article; https://doi.org/10.1172/jci.insight.154940DS1), reflecting higher plasma NEFA and glycerol concentrations per unit fat (Figure 2D), as reported in CD36-KO mice (34). These elevated levels of plasma NEFA and glycerol per gram of adipose suggest increased lipolysis in the Adipo-KO mice than WT mice. Curiously, both *CD36*, a protein that facilitates transport of long chain fatty acids, and perilipin 1 (*PLIN*), a lipid droplet binding protein (34, 35), were found to be reduced in eWAT of the Adipo-KO mice compared with the WT (Figure 2E).

To more directly test lipolysis, we performed an ex vivo lipolysis assay by measuring NEFA and glycerol release over time from eWAT dissected from WT and Adipo-KO mice. We found basal NEFA and glycerol release to be consistently increased from eWAT of Adipo-KO mice compared with WT mice at all time points examined (Figure 2, F and G). Moreover, isoproterenol stimulated lipolysis more robustly in eWAT isolated from Adipo-KO versus WT mice raised on HFD, based on NEFA concentrations in media over time (Figure 2H) and the rate of NEFA production (Figure 2I). As isoproterenol stimulates lipolysis by activating hormone sensitive lipase (HSL), we measured HSL phosphorylation (p-HSL, Ser660) in response to stimulation in WT and Adipo-KO primary adipocytes, in order to examine the underlying mechanism of lipolysis. In this in vitro culture system, consistent with the ex vivo lipolysis results from eWAT (Figure 2, H and I), we observed a striking ~3-fold increase in p-HSL in Adipo-KO primary adipocytes upon stimulation with isoproterenol (Figure 2J).

### Adipose SWELL1 deletion predisposes to developing NAFLD with overnutrition.

**Figure 3:**
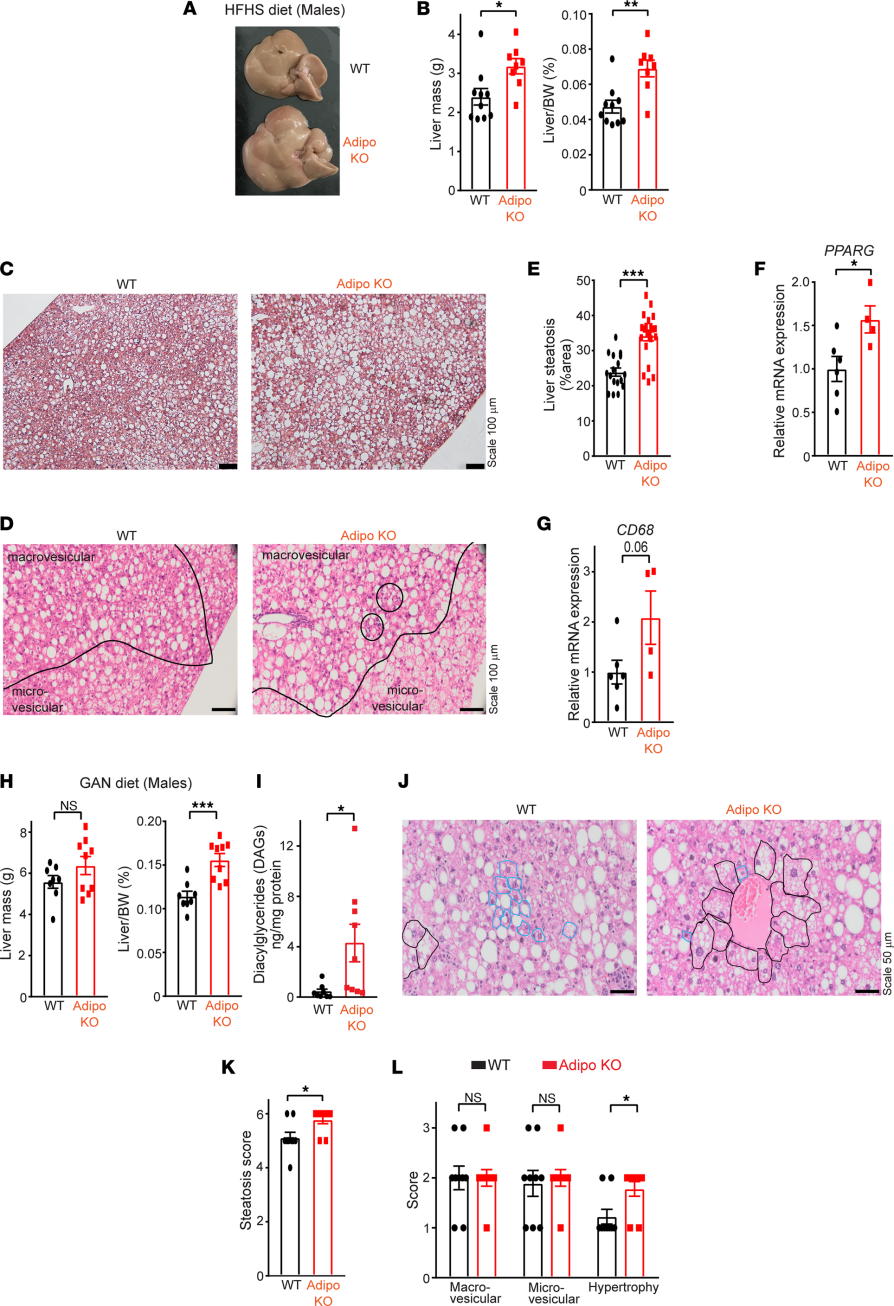
Adipose SWELL1 deletion predisposes to developing NAFLD with overnutrition. (**A**) Representative images of liver dissected from WT (top) and Adipo-KO (bottom) mice (males) fed with HFHS diet for 27 weeks. (**B**) Total liver mass and ratio of liver mass over body weight of WT and Adipo-KO mice from **A**. (**C**) Representative images of H&E-stained liver sections of WT and Adipo-KO mice (Scale bar: 100 μm). (**D**) Micro- and macrovesicular fat regions along with the mononuclear inflammatory cells (solid black circles) in WT and Adipo-KO mice (20× objective, 200× magnification). (**E**) Liver steatosis (%area) estimated from H&E-stained liver sections in **C** of WT (*n* = 2, 17 ROIs) and Adipo-KO (*n* = 2, 21 ROIs) mice using ImageJ software (NIH). (**F**) mRNA expression of *PPARG* in WT (*n* = 6) and Adipo-KO (*n* = 4) livers relative to control *GAPDH*. (**G**) mRNA expression of *CD68* in WT (*n* = 6) and Adipo-KO (*n* = 4) livers relative to control *GAPDH*. (**H**) Total liver mass and ratio of liver mass over body weight of WT (*n* = 8) and Adipo-KO (*n* = 9) mice (males) fed with GAN diet for 23–25 weeks. (**I**) Measurement for total diacylglycerides (DAGs) from WT (*n* = 8) and Adipo-KO (*n* = 9) livers in **H**. (**J**) Representative images of H&E-stained liver sections of GAN diet–fed WT and Adipo-KO mice in **H** indicating cells that are normal (blue) and with hepatocellular hypertrophy (black outline) (40× objective). (**K** and **L**) Cumulative steatosis score (**K**) derived from macrovesicular, microvesicular, and hepatocellular hypertrophy scores (**L**) of liver sections from GAN diet–fed WT and Adipo-KO (*n* = 9 each) mice. Data are represented as mean ± SEM. Two-tailed unpaired *t* test was used in **B**, **E**–**I**, **K**, and **L** where *, **, and *** represent *P* < 0.05, *P* < 0.01, and *P* < 0.001, respectively. ROIs, regions of interest.

Adipose inflammation associated with increased lipolysis and elevated circulating free fatty acids (FFAs) are prone to ectopic lipid deposition in peripheral tissues. Indeed, male Adipo-KO mice raised on HFHS diet were noted to have a significant increase in both total (32%) and normalized (46%) liver mass (Figure 3, A and B). Female Adipo-KO mice showed no differences in these parameters under the same experimental conditions (Supplemental Figure 2, A–C). This may reflect sex differences in the propensity of male versus female mice to develop obesity in response to different diets (36). Histological examination of livers (HFHS diet) from Adipo-KO mice compared with WT revealed the increase in liver mass to be associated with a 43% increase in hepatic steatosis (Figure 3, C–E), including both macro- and microvesicular steatosis (Figure 3D). Consistent with increased hepatic steatosis, peroxisome proliferator–activated receptor γ (*PPARG*) expression, a driver of hepatic steatosis (37, 38), was induced 1.6-fold in Adipo-KO livers (Figure 3F), while other hepatic lipogenic genes, such as adipocyte protein 2 (*AP2*), sterol regulatory element binding protein-1a/1c (*SREBP1A*, *SREBP1C*), acetyl-CoA carboxylase 1 (*ACC1*), stearoyl-CoA desaturase 1 (*SCD1*), and fatty acid synthase (*FAS*), remained unchanged (Supplemental Figure 3A). Moreover, the Adipo-KO livers had more foci of mononuclear inflammatory cells and developed greater lobular inflammation compared with WT upon histological scoring (Figure 3D and Supplemental Figure 3B). Correspondingly, *CD68* expression, a marker of macrophage and monocyte lineage (39, 40), was increased 2-fold in Adipo-KO livers (Figure 3G). Other inflammatory genes, *TLR4*, *TNFA*, *TGFB*, *IL1B*, and *IL6*, were unaltered (Supplemental Figure 3C). Similar to the phenotype observed on HFHS diet, Adipo-KO mice (males) raised on GAN diet exhibited significant increases (36%) in normalized liver mass (Figure 3H). Moreover, the livers from Adipo-KO mice exhibited an approximately 10-fold increase in hepatic diacylglycerides (DAGs; Figure 3I), but no increase in hepatic triacylglyceride (TAG; Supplemental Figure 3D) level was observed compared with the WT livers. Histological examination (Figure 3J) revealed increased hepatic steatosis (Figure 3, J and K) and hepatocellular hypertrophy (Figure 3, J and L) in Adipo-KO mice compared with WT mice raised on a GAN diet.

### Adipose SWELL1 depletion alters the hepatic lipid species profile in HFHS-fed mice.

**Figure 4:**
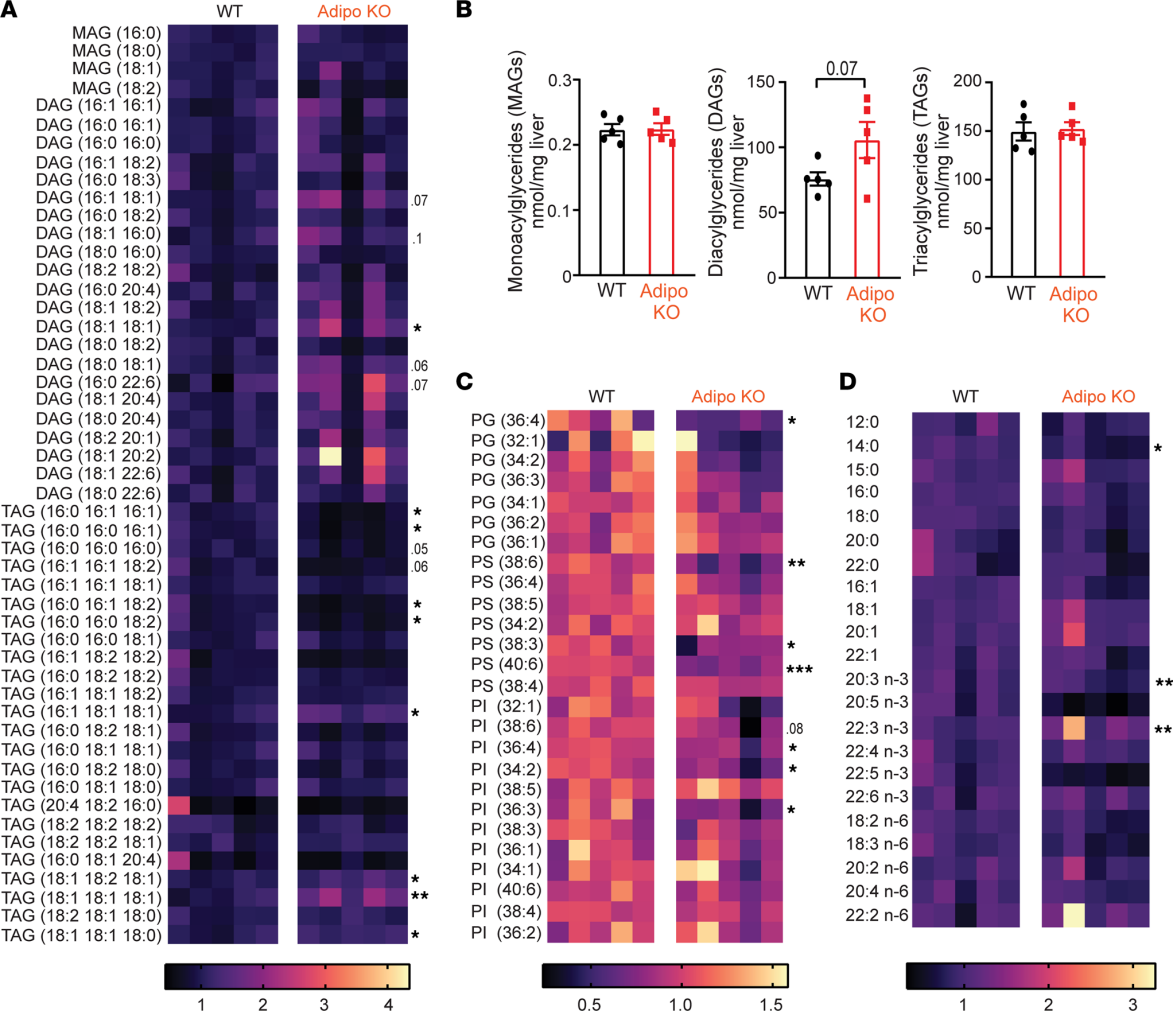
Adipose SWELL1 depletion alters the hepatic lipid species profile. (**A** and **B**) Relative abundance (**A**) and total content of hepatic lipid species for MAGs, DAGs and TAGs (**B**) in Adipo KO compared with WT mice (*n* = 5 each, males) on HFHS diet for 27 weeks. (**C** and **D**) Relative abundance of phosphatidylserine**/**phosphatidylinositol**/**phosphatidylglycerol (**C**) and FFAs (**D**) in Adipo-KO compared with WT mice (*n* = 5 each, males) on HFHS diet for 27 weeks. Two-tailed unpaired *t* test was used in **A**–**D** where *, **, and *** represent *P* < 0.05, *P* < 0.01, and *P* < 0.001, respectively, and *P* values are listed when 0.05 < *P* < 0.1.

Next, we performed a lipidomic screen to measure monoacylglycerides (MAGs), DAGs, TAGs, cholesterol and sphingolipids, and bioactive phospholipids by liquid chromatography/mass spectrometry to examine the lipid species contributing to hepatic steatosis in HFHS-fed Adipo-KO mice. We found that MAGs were unchanged in Adipo-KO livers compared to WT livers (Figure 4A and Supplemental Figure 4A). Remarkably, 18:1/18:1 DAGs (oleic acid incorporated) were increased 64% in Adipo-KO livers compared with WT (Figure 4A and Supplemental Figure 4B), with 18:1-containing DAG lipid species, 16:1/18:1, 18:1/16:0, and 18:0/18:1, showing increasing trends, at 52%, 33%, 37% increased, respectively (Figure 4A and Supplemental Figure 4B), and 16:0/22:6 DAG increased by 76%. All other DAG species were essentially unchanged. Indeed, increases in specifically oleic acid–containing (18:1) DAGs have been shown to drive protein kinase Cε–mediated hepatic insulin resistance in NAFLD (41–43) and also stimulate the androgen receptor to drive obesity and NAFLD-associated hepatocellular cancer (44).

Consistent with increases in 18:1/18:1 DAG, 18:1/18:1/18:1 TAG (oleic acid containing) was also markedly increased 71% in Adipo-KO livers compared with WT, as were 18:1/18:2/18:1, 18:1/18:1/18:0, and 16:1/18:1/18:1 TAGs, at 26%, 18%, and 35%, respectively (Figure 4A and Supplemental Figure 4C). Oleic acid–containing TAGs (18:1) are believed to contribute significantly to hepatic steatosis in NAFLD, with dual modulatory functions for the progression of disease (45). Conversely, 16:0- and 16:1-containing TAGs were generally decreased in Adipo-KO livers (Figure 4A and Supplemental Figure 4C), while all other TAG species were essentially unchanged. Though cumulatively, the total MAGs and TAGs were similar in WT and Adipo-KO livers, the DAGs exhibited an approximately 39% increase (*P* = 0.07) in mice raised on HFHS diet (Figure 4B), similar to that observed in livers of mice raised on GAN diet (Figure 3I). Cholesterol and sphingolipid types including sphingosine, sphinganine, 3-keto-sphinganine, and sphingosine-1-phosphate (S1P) were similar between WT and Adipo KO (Supplemental Figure 4, D and E).

Since dynamic changes in phospholipid (PL) content is another feature in NASH pathogenesis that influences hepatocyte membrane integrity (46), and predisposes to hepatocyte injury, we measured phosphatidylserine (PS), phosphatidylinositol (PI), and phosphatidylglycerol (PG). Of the 7 measured PS subtypes, 38:6, 38:3, and 40:6 were significantly decreased by 34%, 20%, and 25%, respectively, in Adipo-KO livers (Figure 4C and Supplemental Figure 4F). Similarly, PI subtypes 36:4, 34:2, and 36:3 were decreased by 22%, 30%, and 32%, respectively, in Adipo-KO livers (Figure 4C and Supplemental Figure 4G). PG was largely unaltered in WT and Adipo-KO livers, except for 36:4 with a 39% decrease in Adipo KO as compared with WT (Figure 4C and Supplemental Figure 4H). Saturated FFAs were unchanged except for a significant decrease (23%) in myristic acid (14:0) in the Adipo-KO livers (Figure 4D and Supplemental Figure 4I). A lower content (50%) of n-3 polyunsaturated FFAs (n-3 PUFAs) eicosapentaenoic (20:5) and docosapentaenoic acid (22:5) was also observed, while the remaining species were unaltered (Figure 4D and Supplemental Figure 4I). The loss in phospholipids and n-3 FFAs and increases in DAGs and TAGs are consistent with the lipidomic profile of other murine NAFLD models (47, 48).

### Adipose SWELL1-KO mice are predisposed to age-related NAFLD.

**Figure 5:**
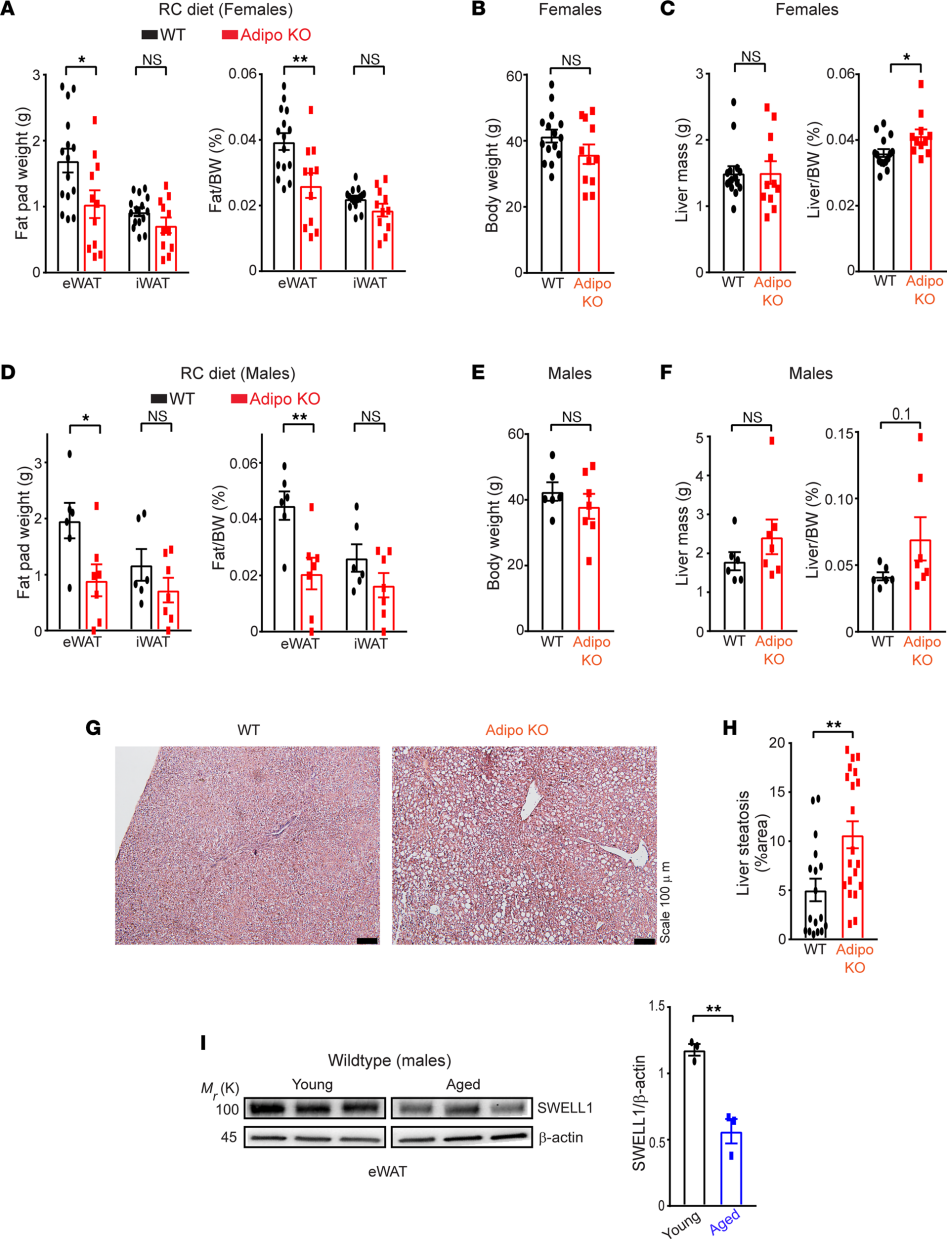
Adipose SWELL1 expression protects against age-related NAFLD and declines with aging. (**A**) Total mass of epididymal (eWAT) and inguinal (iWAT) fat pads and their corresponding ratio of fat pad over body weight, (**B**) total body weight, (**C**) total liver mass, and ratio of liver mass over body weight dissected from WT (*n* = 15) and Adipo-KO (*n* = 11) female mice ~12 months old on RC diet. (**D**) Total mass of epididymal and inguinal fat pads and their corresponding ratio of fat pad over body weight, (**E**) total body weight, (**F**) total liver mass, and ratio of liver mass over body weight dissected from male WT (*n* = 6) and Adipo-KO (*n* = 7) male mice ~18–21 months old on RC diet. (**G**) Representative images of H&E-stained liver sections of WT and Adipo-KO mice from **B** (Scale bar: 100 μm). (**H**) Liver steatosis (%area) estimated from H&E-stained liver sections in **G** of WT (*n* = 2, 17 ROIs) and Adipo-KO (*n* = 2, 20 ROIs) mice using ImageJ software. (**I**) Representative image of Western blot comparing SWELL1 protein expression in epididymal adipose tissue isolated from young (2–3 months old, males) and aged (18–21 months old, males) WT mice fed with RC diet (left) and its corresponding densitometric ratio (right). Data are represented as mean ± SEM. Two-tailed unpaired *t* test was used in **A**–**F**, **H**, and **I** where *, *P* < 0.05, and **, *P* < 0.01.

Similar to mice raised on HFHS diet, both male and female aged Adipo-KO (~12–21 months) raised on an RC diet (18% kcal fat; 59% kcal carbohydrate) developed reduced adiposity compared with WT mice, driven largely by a reduction in eWAT, based on both absolute (39% and 54% in females and males, respectively) and normalized fat pad weights (34% and 54% in females and males, respectively; Figure 5, A and D), with no significant reduction in total body weight (Figure 5, B and E). This impairment in age-related adipose tissue expansion in Adipo-KO mice was associated with a mild, but statistically significant, increase in normalized liver mass in female mice (~14%) (Figure 5C) and a trend toward increased liver mass parameters in males (Figure 5F). Combining these data from both sexes reveals significantly lower fat content and higher liver ratio in aged Adipo-KO mice compared with WT mice (Supplemental Figure 5, A and B). Histological examination of livers from aged Adipo-KO females revealed a significant increase in hepatic steatosis (111%) compared with WT controls (Figure 5, G and H). Taken together, these data suggest that maintaining adipose SWELL1 expression, activity, and signaling is metabolically protective against NAFLD, both in the setting of overnutrition and with aging. Indeed, consistent with this notion, adipose SWELL1 protein expression was significantly higher in young mice (2–3 months old, males) as compared with aged mice (~2-fold, Figure 5I) (~18–21 months old, males), indicating that adipose SWELL1 protein declines with age and may contribute to age-related metabolic dysfunction.

### Male Adipo-KO mice develop HCC with aging.

**Figure 6:**
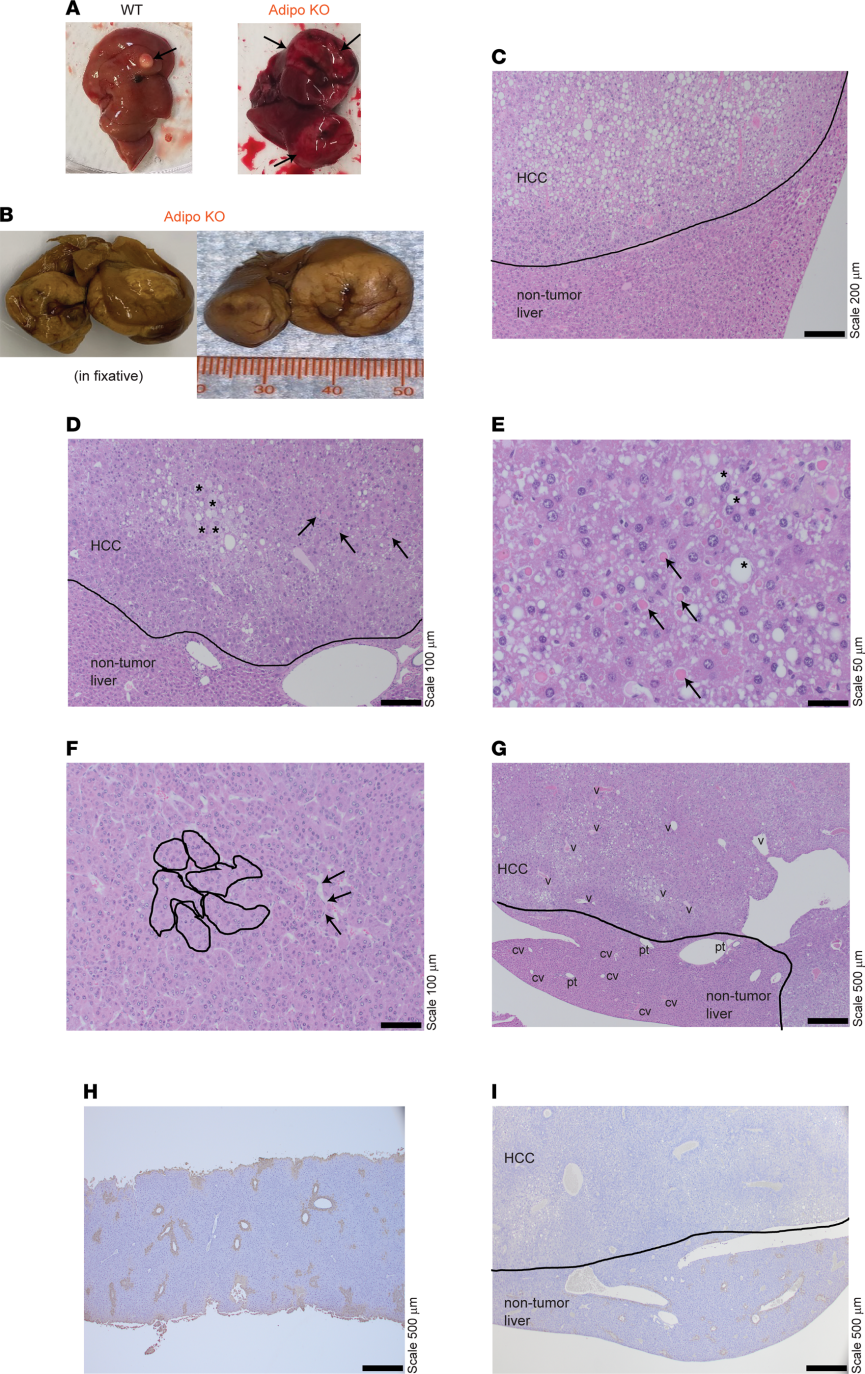
Male adipose SWELL1-KO mice develop HCC with aging. (**A**) Representative images of livers isolated from male WT and Adipo-KO mice with tumors indicated by black arrows. (**B**) Formalin-fixed Adipo-KO liver with massive hepatic tumors. (**C** and **D**) H&E-stained liver sections of Adipo-KO mice with indicated nontumor and tumor-containing regions. * indicates enlarged and pleomorphic cells, and black arrows indicate eosinophilic hyaline globules in **D**. (**E**–**G**) Liver sections of Adipo-KO mouse with HCC morphological features. * indicates large- and intermediate-sized fat droplets, and black arrows indicate eosinophilic hyaline droplets within the HCC cells in **E**. Nested islands of HCC are outlined in solid black lines, and the black arrows indicate the endothelial wrapping around these nests with flattened endothelial cells in **F**. Abnormal veins in HCC regions are indicated with “v,” and normal portal tracts and central veins are indicated as “pt” and “cv” in the nontumorous region in **G**. (**H** and **I**) Representative images of glutamine synthetase staining from a normal WT (**H**) and Adipo-KO (**I**) liver with HCC. Each is a 10× eyepiece with a 4×, 10×, 20×, 40× objective; 40× magnification for images in **G**–**I**; 100× magnification for image in **C**; 200× magnification for images in **D** and **F**; and 400× magnification for image in **E**.

Chronic NAFLD and inflammation associated with NASH predisposes to HCC and occurs predominantly in males (49). Remarkably, 3/7 of the aged Adipo-KO males had massive tumors on gross inspection, as compared with 1/6 WT males, which had a small tumor (Figure 6, A and B). Further examination of these Adipo-KO livers by H&E staining revealed increased steatosis, particularly in the tumor region (Figure 6C), as compared with the nontumorous region. Gross steatosis and inflammation were similar between WT and Adipo KO (Supplemental Figure 5C). Consistent with HCC, histological features of Adipo-KO livers contained enlarged pleomorphic cells along with eosinophilic hyaline globules (Figure 6, D and E). HCC regions had diagnostic features of malignant hepatocellular proliferation, including nested islands of hepatocytes with peripheral wrapping of flattened endothelial cells along the irregular sinusoidal spaces (Figure 6F). Furthermore, they were devoid of portal structures and included irregularly distributed dilated veins compared with nontumorous regions (Figure 6G). To examine the nature of the tumors, we stained the livers with glutamine synthetase, an enzyme with distinct central perivenular distribution in normal liver that typically has an abnormal distribution in HCC (Figure 6, H and I) (50). Indeed, the aged Adipo-KO livers exhibited an abnormal glutamine synthetase (GS) stain pattern (complete loss) in the tumor region compared with the normal adjacent nontumor region (Figure 6I). The WT group demonstrated normal GS staining (Figure 6H). Taken together, these data reveal that adipose SWELL1 ablation not only impairs healthy adipose depot expansion in the setting of overnutrition (28), and with sedentary aging, but also predisposes to NAFLD, NASH, and consequent HCC associated with longstanding metabolic syndrome.

## Discussion

Adipocytes function, in part, by storing energy as fat to maintain energy homeostasis (51). During nutritional excess, energy is stored in the form of triglycerides, and during caloric deficit, lipolysis is activated to release FFAs and glycerol as energy supply to other cells (52). Adipocytes also function by secreting various cytokines that have autocrine, paracrine, and endocrine effects, thereby regulating metabolic functions of other tissues and organs (52). It has been proposed that adipocytes respond to mechanical stress from lipid expansion and external ECM complexes to regulate signaling events that influence growth, proliferation, and differentiation (20–22, 53). For example, collagen VI (Col VI) is a major component of ECM that normally physically constrains adipocyte expansion. Genetic deletion of Col VI results in larger adipocytes but without increased inflammation and insulin resistance (54). Conversely, chemical inhibition or genetic ablation of matrix metalloproteinase in mice, which increases fibrillar collagen, results in smaller adipocyte size but also without insulin resistance and adipose inflammation (55, 56). Finally, it has been demonstrated in human adipocytes using a 3D culture system that numerous intracellular signaling pathways, including lipolytic pathways and fibro-inflammation, can be activated by mechanical compression (23). In sum, these studies provide convergent evidence that adipocyte development and function are sensitive to mechanical forces.

We recently identified a potentially novel growth pathway in adipocytes mediated by a mechanosensitive VRAC complex encoded by LRRC8a, also known as SWELL1 (24, 28). SWELL1 is a required component of the VRAC complex that upon mechanical stimulation (positive pressure or hypotonic swelling) activates a characteristic I_Cl,SWELL_ current in adipocytes and other cell types (28, 57–60). SWELL1 protein levels are upregulated with obesity, and the I_Cl,SWELL_ is spontaneously activated in adipocytes isolated from obese humans and mice when compared with nonobese controls. We surmise that this SWELL1 activation promotes lipogenesis via insulin/PI3K/AKT2 signaling axis and potentiates adipocyte growth in a feed-forward amplifier manner during caloric excess (24, 28, 29).

In this study, we further examined the implications of adipose-specific SWELL1 ablation (Adipo KO) in diet-induced NAFLD/NASH models and aging. Adipo-KO mice raised on a NAFLD/NASH diet had impaired adipose tissue expansion and increased lipolysis compared with WT mice, contributing to increased ectopic hepatic lipid deposition, exacerbated hepatomegaly, hepatosteatosis, and injury, leading to NAFLD. These data support the notion that adipocyte mechanosignaling is an important regulator of lipid homeostasis. There are examples of other mechanoresponsive or mechanosensitive ion channels expressed in adipocytes that regulate systemic metabolism. TRPV4 is a broadly expressed polymodally activated cation channel that can be activated by mechanical stimuli, including stretch and swelling (61). In adipose, TRPV4 negatively regulates PPARγ coactivator 1α, a key modulator of mitochondrial biogenesis and oxidative metabolism (25). TRPV4 activates a proinflammatory response in adipose, and genetic ablation of TRPV4 leads to metabolically healthy mice with improved insulin sensitivity that are resistant to diet-induced obesity (DIO). Such targets are beneficial in drug development, and indeed pharmacological targeting of TRPV4 by antagonist GSK205 improved glucose homeostasis in DIO mice (25). Piezo1 is another mechanosensitive ion channel (62) that is broadly expressed, not only in classically mechanoresponsive tissues such as endothelium, where it regulates blood pressure and vascular development (63–67), but also in adipose tissue. Specifically, in adipose, Piezo1 has differential effects depending on the timing of the gene inactivation (26, 27). In mature adipocytes, loss of Piezo1 promotes adiposity by increasing adipocyte volume with overnutrition, though this is partially offset by a reduction in adipocyte number (26). Piezo1 is also required for adipocyte differentiation wherein precursor cells are recruited and a differentiation cascade is initiated in a fibroblast growth factor 21–dependent manner (26). In contrast, constitutive Piezo1 depletion reduces perigonadal fat mass and mildly decreases adipocyte size (27). Irrespective of the timing, Piezo1 gene inactivation increases inflammation, glucose intolerance, and insulin resistance in DIO mice. Interestingly, similar to the Adipo-KO mice, early deletion of adipocyte Piezo1 also increases lipolysis, hepatic inflammation, and triglycerides, contributing to exacerbated NAFLD (27). Activation of lipolysis occurring via p-HSL has been demonstrated in adipocytes and adipose tissue explants (68). Likewise, Adipo-KO mice exhibited evidence of increased lipolysis (NEFAs and glycerol) in vivo and ex vivo, associated with increased HSL activation in vitro. Our results and data from TRPV4 and Piezo1 mouse models support the overarching notion that adipocyte mechano-signaling regulates systemic metabolism, especially under conditions of overnutrition.

Hepatic lipid species composition has been characterized in hepatic steatosis (47, 69, 70). Increased DAGs are believed to contribute to NAFLD progression, and their interaction with protein kinase Cε can lead to NAFLD-associated hepatic insulin resistance (41–43). Indeed, we specifically observed increases in DAG content in Adipo-KO livers under 2 diet types (HFHS and GAN) while the MAG and TAG content remained unchanged. This specific accumulation of DAGs dependent on SWELL1 is intriguing and warrants further exploration. Among all the DAGs, increases in particularly oleic acid (36:2) species were observed. Oleic acid (36:2) has been reported to be elevated with hepatic lipogenesis and also shown to activate androgen receptor–mediated AKT stimulation, a putative pathway linked to higher risk of HCC development in obese patients with NAFLD, with a male sex predominance (44). Dynamic PL remodeling is another feature in NAFLD pathogenesis that targets the hepatocyte membrane integrity (46). Decrease in PLs, such as PS, PI, phosphatidylcholine, and phosphatidylethanolamine, correlates with NAFLD progression (48, 69, 71), and treatment with supplemental PLs alleviates steatosis (72, 73). Indeed, Adipo-KO mice raised on HFHS diet also exhibited decreased hepatic PS and PI content and accelerated NAFLD. Also, consistent with the reductions in hepatic n-3 PUFAs observed in the Adipo-KO mice, diminished n-3 PUFAs have been attributed to NAFLD pathogenesis (74, 75), and their supplementation in diet has shown to alleviate NAFLD by suppressing the sterol regulatory element binding protein-1–mediated lipogenesis, inflammation, and oxidative stress (76–80).

Curiously, we found that Adipo-KO mice exhibited impairments in epididymal adipose tissue expansion and developed hepatic steatosis with aging alone when raised on an RC diet. These data suggest that adipose SWELL1 is protective against NAFLD not only in the setting of overnutrition, but also with aging. In addition to age-related hepatic steatosis, we observed an increase in the frequency of HCC in aged male Adipo-KO mice, as compared with littermate controls. These tumors had complete loss of GS staining correlating with pathology of HCC (50). Consistent with impairments in adipose insulin sensitivity as a putative mechanism for HCC in Adipo-KO mice, the fat-specific insulin receptor knockout (FIRKO) or FIRKO in combination with insulin-like growth factor-1 receptor–KO mice also exhibit lipodystrophic phenotypes, and similarly, develop liver tumors with aging (81, 82). These KO models are diabetic and develop hepatic steatosis by 3 months of age in the absence of inflammation and fibrosis. Upon aging to a year, hepatic steatosis along with chronic lipotoxic conditions drive liver disease to the point of developing severely dysplastic nodules (81).

In summary, the adipose SWELL1-KO mouse model provides potentially unique conceptual insights into the relationship between a mechanoresponsive ion channel in adipocytes and maintenance of healthy adipose function to prevent dysregulated lipid homeostasis with overnutrition and aging. The metabolic dysregulation that ensues from adipose SWELL1 depletion drives the full spectrum of liver disease from hepatic steatosis to HCC. These findings also suggest that pharmacological modulation of adipose SWELL1 may provide a novel approach for the treatment of NAFLD (83) and progression to HCC.

## Methods

### Animals.

*SWELL1^fl/fl^* mice were established in our previous work (28). Adipose-specific SWELL1 KO (Adipo KO) was generated by crossing Adiponectin-Cre recombinase driver–containing C57BL/6 mice (Charles River Laboratory) with the *SWELL1^fl/fl^* mouse as described previously (28). The WT (*SWELL1^fl/fl^*) and Adipo-KO mice were fed ad libitum with RC diet (18% kcal fat; 59% kcal carbohydrate) with free access to water and switched to HFHS diet (Research Diets, Inc., 58% kcal fat, 18% sucrose; D12231), HFD (Research Diets, Inc., 60% kcal fat, 20% kcal carbohydrate; D12492), or GAN diet (Research Diets, Inc., 40% kcal fat, 40% kcal carbohydrate; D09100310) at 7–9 weeks of age. In 1 batch of mice, the GAN diet regimen was started at 3–6 months of age and maintained for 22 weeks. Aged mice were maintained on RC diet. Sex of the mice used in the studies are indicated in the descriptions in Results. Investigators followed a blinded protocol for all the experiments and subsequent analysis.

### Tissue isolation.

The mice were anesthetized by injecting Avertin (0.0125 g/mL in H_2_O) or with 1%–4% isoflurane followed by cervical dislocation. Livers were removed, weighed, and fixed in 10% neutral buffered formalin and paraffin-embedded for further histology. The remaining tissue was frozen in liquid nitrogen for RNA and protein. The fat pads were isolated and stored similarly.

### Body composition.

Body composition of mice maintained on RC diet, HFHS diet, and HFD was estimated using Bruker’s Minispec LF50 NMR machine. Mice maintained on HFD for 6–9 months were switched to RC diet for 4 weeks, and the body composition was measured at days 0, 11, 14, 21, and 28 by NMR. Body composition of mice maintained on GAN diet was measured using EchoMRI 3-in-1 Body Composition Analyzer.

### Lipolysis.

Epididymal fat pads were excised from the mice (11- to 12-week-old females on RC diet in the case of basal stimulation; 14-week-old males on HFD for 5 weeks in the case of 50 nM isoproterenol stimulation) and stored in phenol red–free DMEM (Gibco) containing 2% fatty acid–free BSA (Roche). The fat pads were kept in media at 37°C until all the fat pads were removed from study mice. Excess liquid from the fat was quickly removed by blotting on a paper, and fat was cut into pieces of around 10–30 mg each. Each piece was recorded for weight and then placed in a 24-well plate containing 250 μL media and further cut down into 1–3 mm pieces with scissors. Equal volume of media containing vehicle or isoproterenol (final concentration of 50 nM) was added to the well, and a 50 μL aliquot was collected at 30, 60, 90, and 120 minutes time points. The extent of lipolysis was estimated by measuring glycerol (Free Glycerol kit, MilliporeSigma – F6428) and nonesterified FFAs [FujiFilm HR Series NEFA-HR (2) kits] and normalizing to the recorded fat weight in the above step. Briefly, for the glycerol assay, 25 μL of the sample and standard (MilliporeSigma – G7793) were added to a 96-well plate followed by the addition of 100 μL of glycerol reagent. After incubation at 37°C for 5 minutes, the absorbance was measured at 540 nm. For the NEFA assay, 10 μL of the sample was added to a 96-well plate followed by the addition of 100 μL of Solution A and incubated at 37°C for 5 minutes. Then a 50 μL of Solution B was added and incubated at 37°C for 5 minutes. The final absorbance was measured at 550 nm. Random plasma glycerol was measured by adding 10 μL of the sample and standard to a 96-well plate followed by the addition of 100 μL of glycerol reagent. After incubation at 37°C for 5 minutes, the absorbance was measured at 540 nm. Random/fasting plasma NEFAs were measured using the same above kits as per the manufacturers’ instructions.

### Primary cell isolation and culture.

The inguinal fat pad was excised from the mouse and washed with Hanks’ balanced salt solution (HBSS). The tissue was minced well with a razor blade and further homogenized in digestion buffer (HBSS + 3% BSA, 1 mM CaCl_2_, 1 mM MgCl_2_, 8 mg/mL collagenase D from Roche, 2.4 U/mL Dispase II from Roche) for 30 minutes at room temperature (RT) with rotation every 10 minutes. The suspension was then filtered through a 100 μm cell strainer (VWR) and mixed with equal volumes of culture medium (DMEM F12 + 10% FBS + 1% penicillin/streptomycin). The cells were spun at 400*g* for 5 minutes at room temperature and the supernatant was discarded. The wash cycle was repeated 2 more times, and the resulting stromal vascular fraction (SVF) cells were plated on a 10 cm dish coated with collagen type I (56 μg/μL). After 24 hours, the cells were washed with 1× PBS and were maintained in culture medium. Upon reaching 70% confluence, either adenovirus CMV-GFP or CMV-cre-GFP (both from University of Iowa, Viral Vector Core) was added to the cells in low serum media (DMEM F12 + 2% FBS + 1% penicillin/streptomycin). The cells were maintained in the culture media with virus for 48 hours and replaced with culture medium. After 48 hours, the media were replaced with differentiation media (culture medium + 850 nM insulin + 0.5 mM IBMX + 1 μM dexamethasone + 1 μM rosiglitazone) and differentiated for 9 days. On day 9 of differentiation, cells were starved in no serum culture medium for 6 hours and stimulated with 100 nM isoproterenol-containing culture medium for 15 minutes. The cells were then washed with ice-cold 1× PBS and lysed in 1× RIPA buffer with protease and phosphatase inhibitors.

### Liver DAG and TAG assay.

Ice-cold saline solution (~500 μL) was added to 80–100 mg of liver in a 1.5 mL safe-lock microcentrifuge tube. A few pieces of stainless steel beads (Next Advance, SSB32) were added to the tube and homogenized with level 8 for 3 minutes in Disrupter (Next Advance, Bullet Blender Blue 24). DAG measurement was carried out using the Diacylglycerol (DAG) ELISA Kit (Abbexa, 258320) as per the manufacturer’s instructions. Briefly, liver homogenates were added to a 96-well plate and incubated with detection reagent A for 1 hour at 37°C. After a wash step 3 times, detection reagent B for was added for another 30 minutes at 37°C. After a wash, the plate was further incubated with the substrate 3,3′5,5′-tetramethylbenzidine (part of the Diacylglycerol [DAG] ELISA Kit from Abbexa) for 20 minutes at 37°C, and the reaction was terminated using stop buffer. For TAG assay, 100 μL of liver homogenate was mixed with equal volume of 1% sodium deoxycholate (MilliporeSigma, D6750-10G) solution. The resulting mixture was vortexed for 30 seconds and incubated for 5 minutes at 37°C. After a brief vortex, 5 μL of liver homogenates was added in a 96-well plate followed by 150 μL of Infinity TG reagent (Thermo Fisher Scientific, TR22421). The plate was further incubated at 37°C for 5 minutes along with the Triglyceride Standard (Pointe Scientific, T7531-STD). In both cases, the absorbance was read at 450 nm and estimated for concentrations from the standard curve. Protein estimation was carried out by using *DC* Protein Assay Reagent A/B/S (Bio-Rad, 5000115, 5000114) for DAG and TAG normalization.

### Histology and scoring.

H&E and GS staining were carried out on the sections from the paraffin-embedded liver and were further imaged using Olympus BX-61 and BX-51 microscopes. The steatosis area percentage was estimated using ImageJ by selecting 17–22 ROIs per group with a lower and upper area threshold of 115 and 255 (84). NAFLD activity scoring for steatosis and lobular inflammation was estimated as per the guidelines for NAFLD scoring in rodent models (85).

### Western blot.

Primary adipocytes derived from SVF cells were lysed in RIPA buffer (150 mM NaCl, 1 mM EDTA, 1 mM EGTA, 1% NP-40, 0.5% sodium deoxycholate, 0.1% SDS, 50 mM HEPES, pH 7.4) with proteinase/phosphatase inhibitors (Roche). Fat tissues from aging mice were homogenized in RIPA buffer with the following composition: 150 mM NaCl, 20 mM HEPES, 1% NP-40, 5 mM EDTA, pH 7.4. Further lysis was carried out by sonicating for 10-second intervals for 3 cycles. The supernatant was collected by centrifugation at 19,000*g* for 20 minutes at 4°C that was repeated 1 more time to remove excess fat and further estimated for protein concentration using *DC* Protein Assay kit (Bio-Rad). The protein samples were prepared in 4× laemmli buffer (Bio-Rad), and approximately 10–15 μg of protein was loaded in a 4%–15% gradient gel (Bio-Rad). Proteins were then transferred onto the PVDF membranes and blocked in 5% BSA (or 5% milk for SWELL1) in TBST buffer (0.2 M Tris, 1.37 M NaCl, 0.2% Tween-20, pH 7.4) for 1 hour and incubated with appropriate primary antibodies (5% BSA or 5% milk for SWELL1) overnight at 4°C. For secondary antibody, goat anti-rabbit (Bio-Rad) in 1% BSA (or 1% milk for SWELL1) in TBST buffer was added to the membrane for 1 hour at RT and further developed by chemiluminescence (Pierce) and visualized using a Chemidoc imaging system (Bio-Rad). ImageJ software was used for estimating densitometric ratios. The following primary antibodies were used: anti–β-actin (8457s), anti–p-HSL (Ser660, 45804s), and anti-HSL (4107S) from Cell Signaling Technology and anti-SWELL1 as previously described (86). See complete unedited blots in the supplemental material.

### RNA isolation and quantitative real-time PCR.

Frozen liver and eWAT samples were homogenized in TRIzol (Invitrogen) reagent. Total RNA isolation was carried out using PureLink RNA kit (Life Technologies) as per the manufacturer’s protocol, and concentration and purity of the RNA were estimated by NanoDrop instrument (Thermo Fisher Scientific). A total of 1 μg of RNA was used to amplify cDNA using iScript Reverse Transcriptase kit (Bio-Rad). Quantitative real-time PCR was carried out by adding 0.3–0.5 μL of cDNA per well with either Power SYBR Green PCR Master Mix (Applied Biosystems) or iTaq Universal SYBR Green Supermix (Bio-Rad). The following primers were used (5′ to 3′): *PPARG* forward: CTTGTGAAGGATGCAAGGGT and reverse: ATACAAATGCTTTGCCAGGG, *AP2* forward: TGGGAACCTGGAAGCTTGTCTC and reverse: GAATTCCACGCCCAGTTTGA, *FAS* forward: AGCACTGCCTTCGGTTCAGTC and reverse: AAGAGCTGTGGAGGCCACTTG, *CD36* forward: GCTGTGTTTGGAGGCATTCT and reverse: CCTTGATTTTGCTGCTGTTC, *PLIN* forward: CTGTGTGCAATGCCTATGAGA and reverse: CTGGAGGGTATTGAAGAGCCG, *TNFA* forward: CATCTTCTCAAAATTCGAGTGACAA and reverse: TGGGAGTAGACACAAGGTACAACCC, *IL-1B* forward: AAGGAGAACCAAGCAACGACAAAA and reverse: TGGGGAACTCTGCAGACTCAAACT, *CD68* forward: CCAATTCAGGGTGGAAGAAA and reverse: CTCGGGCTCGATGTAGGTC, *TLR4* forward: ATAGCTTCTCCAATTTTTCAGAA and reverse: CCATGCCATGCCTTGTCTTCAA, *IL-6* forward: TCCTACCCCAATTTCCAATG and reverse: GGTTTGCCGAGTAGATCTCAA, *TGFB* forward: AAGTTGGCATGGTAGCCCTT and reverse: GCCCTGGATACCAACTATTGC and *GAPDH* forward: TGCACCACCAACTGCTTAG and reverse: GATGCAGGGATGATGTTC. The amplification was carried out in Applied Biosystems StepOnePlus or Bio-Rad real-time PCR system. Measurements were performed in triplicates, and ΔC_T_ calculations were estimated relative to GAPDH as internal standard.

### Hepatic lipidomics.

The mouse liver was homogenized in 2% CHAPS solution (4 mL/g liver). All the lipids were extracted from 50 μL of homogenate using protein precipitation method. The MAG, DAG, and TAG were further extracted with modified Bligh-Dyer method. PS(14:0–14:0) (6 μg/sample) for PS; PI(16:0–16:0) (4.8 μg/sample) for PI; PG(15:0–15:0) (1 μg/sample) for PG; TAG (17:1–17:1–17:1) (50 μg/sample) for TAG; DAG(21:0–21:0) (10 mg/sample) for DAG; MAG(15:0) (5 μg/sample) for MAG; d7-cholesterol (30 μg/sample) for cholesterol, d4-FFA(16:0) for FFA; d7-sphingosine (0.05 μg/sample) for sphingosine, sphinganine, and 3-ketosphinganine; and d7-S1P (0.05 μg/sample) for S1P and sphinganine-1-phosphate (S’1P) were used as internal standards. Internal standards were added to the samples before extraction. Cholesterol and FFA were derivatized with nicotinic acid and 4-aminomethylphenylpyridium, respectively, to improve mass spectrometric sensitivity. Three reagent blank samples were also prepared for FFA, and the means of FFA in reagent blank samples were subtracted from data of study samples. Quality control (QC) samples were prepared by pooling the aliquots of the study samples and injected every 5 study samples to monitor the instrument stability. Only the lipid species with coefficient of variation < 15% in QC samples were reported. The relative quantification of lipids was provided, and the data were obtained as the peak area ratios of the analytes to the corresponding internal standards. The relative quantification data generated in the same batch are appropriate to compare the change of an analyte in a test sample relative to other samples (e.g., control vs. treated, or samples in a time-course study). Under the assumptions that recoveries and mass spectrometric responses of analytes and internal standards are same, the peak area ratio was converted to nmol/mg liver.

Measurement of sphingosine, sphinganine, S1P, S’1P, 3-ketosphingnanine, TAG, DAG, and MAG was performed with a Shimadzu 20AD UFLC system and a Shimadzu SIL-20AC HT autosampler coupled to an API4000 mass spectrometer operated in multiple reaction monitoring mode under electrospray ionization(+). Data processing was conducted with Analyst 1.6.3. Measurement of PS, PG, PI, FFA, and cholesterol was performed with a Shimadzu 10AD HPLC system and a Shimadzu SIL-20AC HT autosampler coupled to a TSQ Quantum Ultra mass spectrometer operated in selective reaction monitoring mode under electrospray ionization(+). Data processing was conducted with Xcalibur 2.0.7. The full lipidomics data are provided as Supplemental Data 1.

### Statistics.

Standard unpaired 2-tailed *t* test and 1- or 2-way ANOVA were performed while comparing 1 or 2 groups. A *P* value less than 0.05 was considered statistically significant. All data are represented as mean ± SEM. All statistical analyses are indicated in the respective legends.

### Study approval.

All mouse studies were carried out in accordance with the approvals from the Institutional Animal Care and Use Committee at the University of Iowa, Iowa City, Iowa, USA, and Washington University at St. Louis, St. Louis. Experimental procedures involving recombinant viruses were performed with the approval of and by the guidelines stipulated by the Institutional Biosafety Committee at the University of Iowa, and the Institutional Biological & Chemical Safety Committee of Washington University at St. Louis.

## Author contributions

Conceptualization was performed by RS; methodology was developed by SKG, JH, DHC, AK, HZ, LX, JDS, and RS; formal analysis was performed by RS, SKG, JH, DHC, AK, and LX; investigation was performed by RS, JH, SKG, DHC, HZ, AK, and LX; RS provided resources; RS, JH, and SKG wrote the original draft; RS, SKG, JH, and JDS reviewed and edited the draft; RS, SKG, JH, DHC, AK, and LX visualized data; RS and JDS supervised the study; and RS acquired funding.

## Supplementary Material

Supplemental data

Supplemental data set 1

## Version 1 02/07/2023

In-Press Preview

## Version 2 03/08/2023

Electronic publication
