# Supplementary material for: Adipose-targeted SWELL1 deletion exacerbates obesity- and age-related nonalcoholic fatty liver disease
Source: JCI Insight. 2023 Mar 8;8(5):e154940. doi: 10.1172/jci.insight.154940 (PMC10077479; doi:10.1172/jci.insight.154940)
Supplement: Supplemental data [file jciinsight-8-154940-s105.pdf]

Adipose-targeted SWELL1 deletion exacerbates obesity- and age-related non-alcoholic fatty liver disease

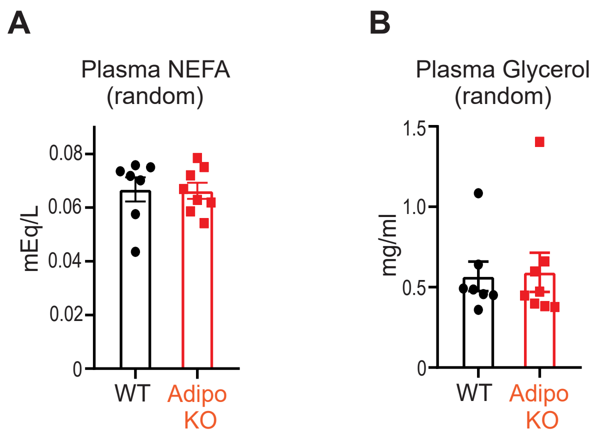

Supplementary Figure 1

Supplementary Figure 1. Plasma composition in male WT and Adipo KO mice under GAN diet. (A-B) Random plasma concentrations for NEFAs **(A)** and glycerol **(B)** in WT (n=7) and Adipo KO (n=8) mice (males) fed with GAN diet for 22 weeks. Data are represented as Mean ±SEM. Two-tailed unpaired t-test was used in **A-B**.

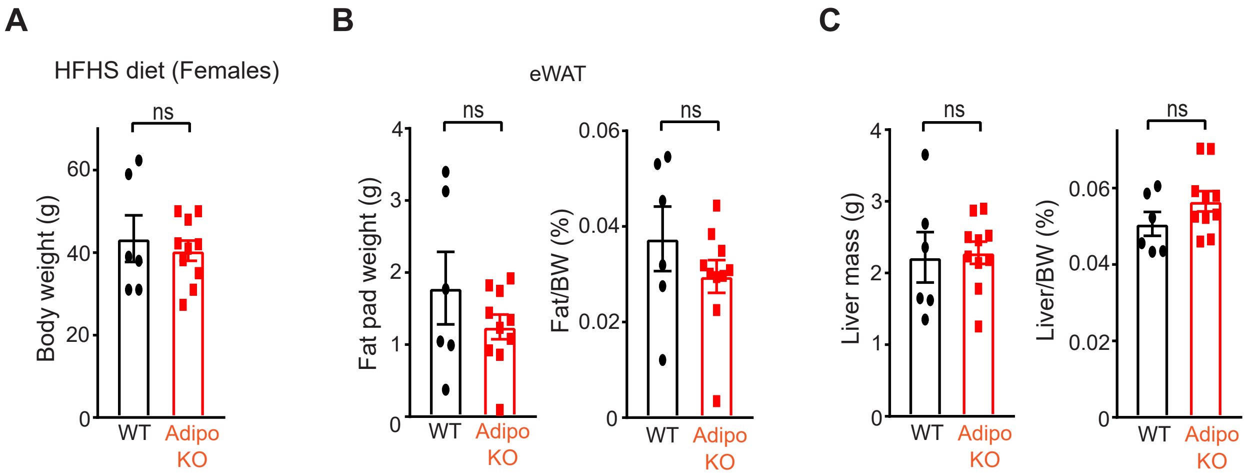

Supplementary Figure 2

Supplementary Figure 2. Body weight, adiposity and liver weights in female WT and Adipo KO mice under HFHS diet. (A) Total body weight of WT (n=6) and Adipo KO (n=10) female mice fed with high-fat/high-sucrose (HFHS) diet for 32 weeks. **(B)** Fat pad weights and ratio of fat pad over body weight of WT and Adipo KO mice from **A**. **(C)** Total liver mass and ratio of liver mass over body weight of WT and Adipo KO mice from **A**. Data are represented as Mean ±SEM. Two-tailed unpaired t-test was used in **A-C**, ns- no significance.

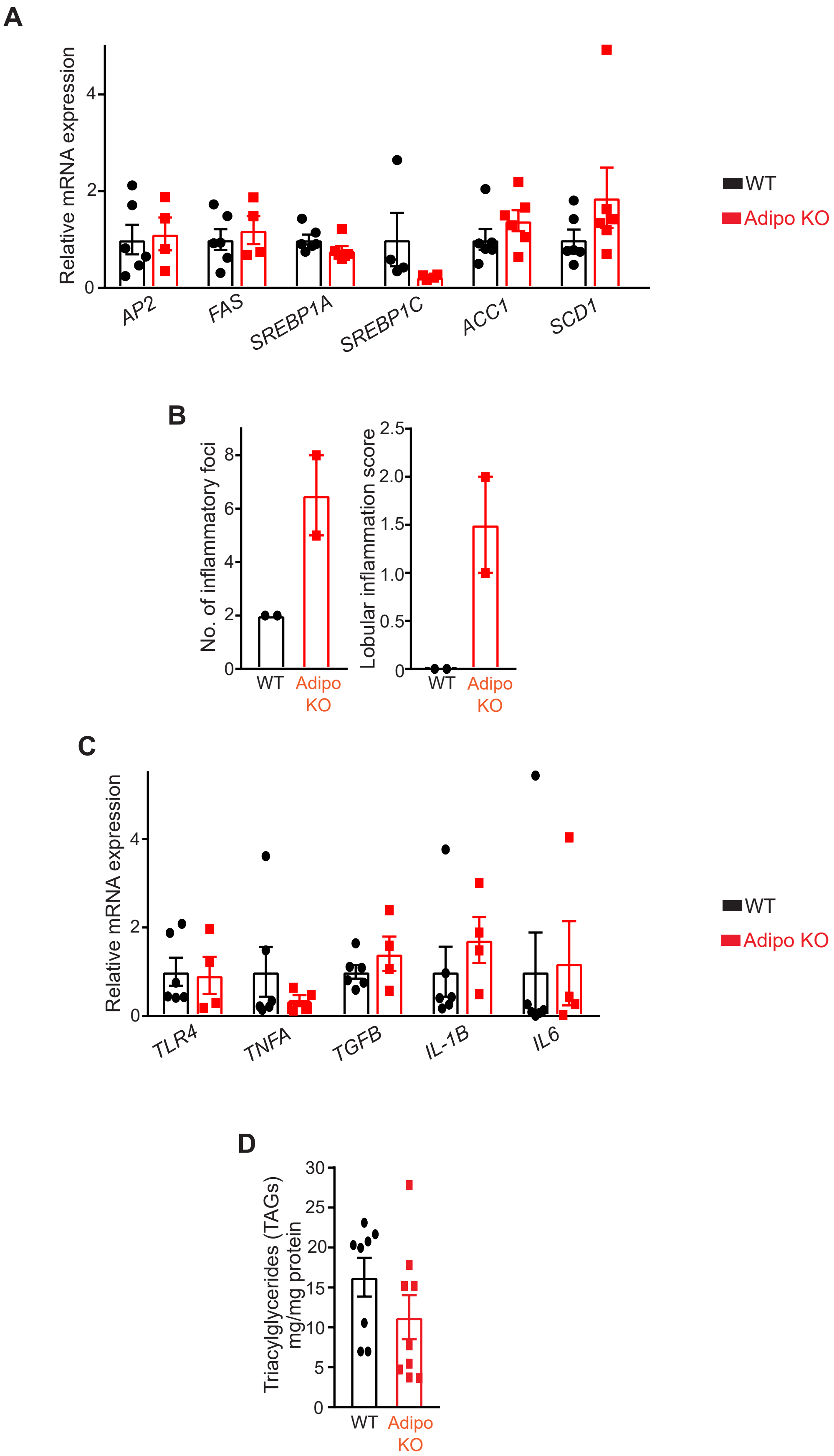

Supplementary Figure 3

Supplementary Figure 3. Hepatic lipogenesis and inflammation in male Adipo KO mice. (A) mRNA expression of hepatic lipogenic genes in WT and Adipo KO (n=4-6) livers relative to control *GAPDH*. **(B)** Estimation of total number of inflammatory foci and the corresponding lobular inflammation score from WT and Adipo KO (n=2) mice from **Figure 3C,D**. **(C)** mRNA expression of hepatic inflammatory genes in WT and Adipo KO (n=4-6) livers relative to control *GAPDH*. **(D)** Estimation of total hepatic triacylglycerides (TAG) from WT (n=8) and Adipo KO (n=9) mice (males) fed with Gubra Amylin NASH (GAN) diet for 23-25 weeks. Data are represented as Mean ±SEM. Two-tailed unpaired t-test was used in **A-D**.

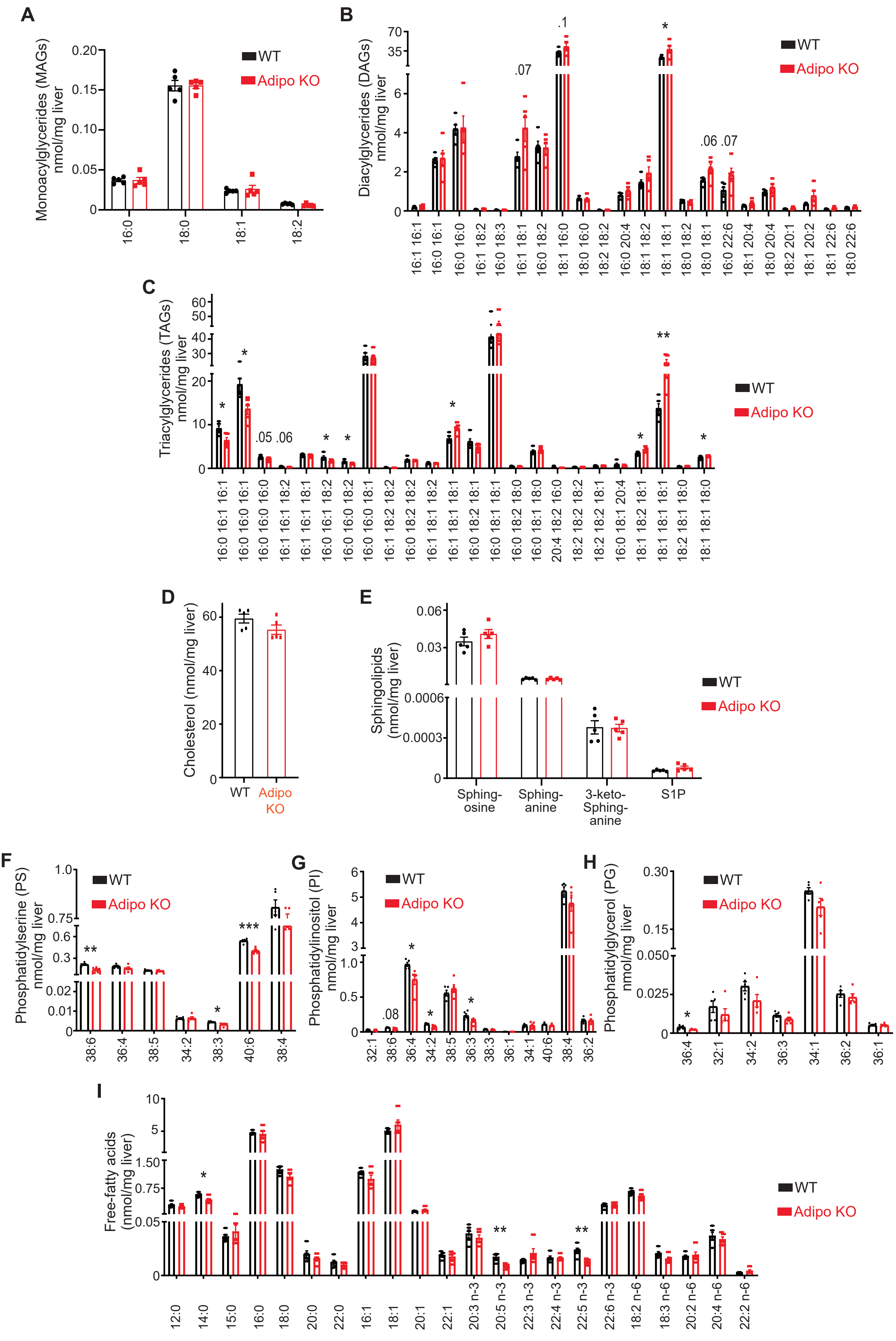

Supplementary Figure 4

Supplementary Figure 4. Hepatic lipid species in WT and Adipo KO mice. (A-I) Hepatic lipid composition in MAGs **(A)**, DAGs **(B),** TAGs **(C)**, cholesterol **(D)**, sphingolipids **(E)**, phosphatidylserine **(F)**, phosphatidylinositol **(G)**, phosphatidylglycerol **(H)** and free-fatty acids **(I)** in WT and Adipo KO mice (n=5 each, males) on high-fat/high-sucrose (HFHS) diet for 27 weeks quantified using LC/MS method. Two-tailed unpaired t-test was used in **A-I** where *, ** and *** represents $p<0.05$, $p<0.01$ and $p<0.001$ respectively and p values listed when $0.05<p<0.1$.

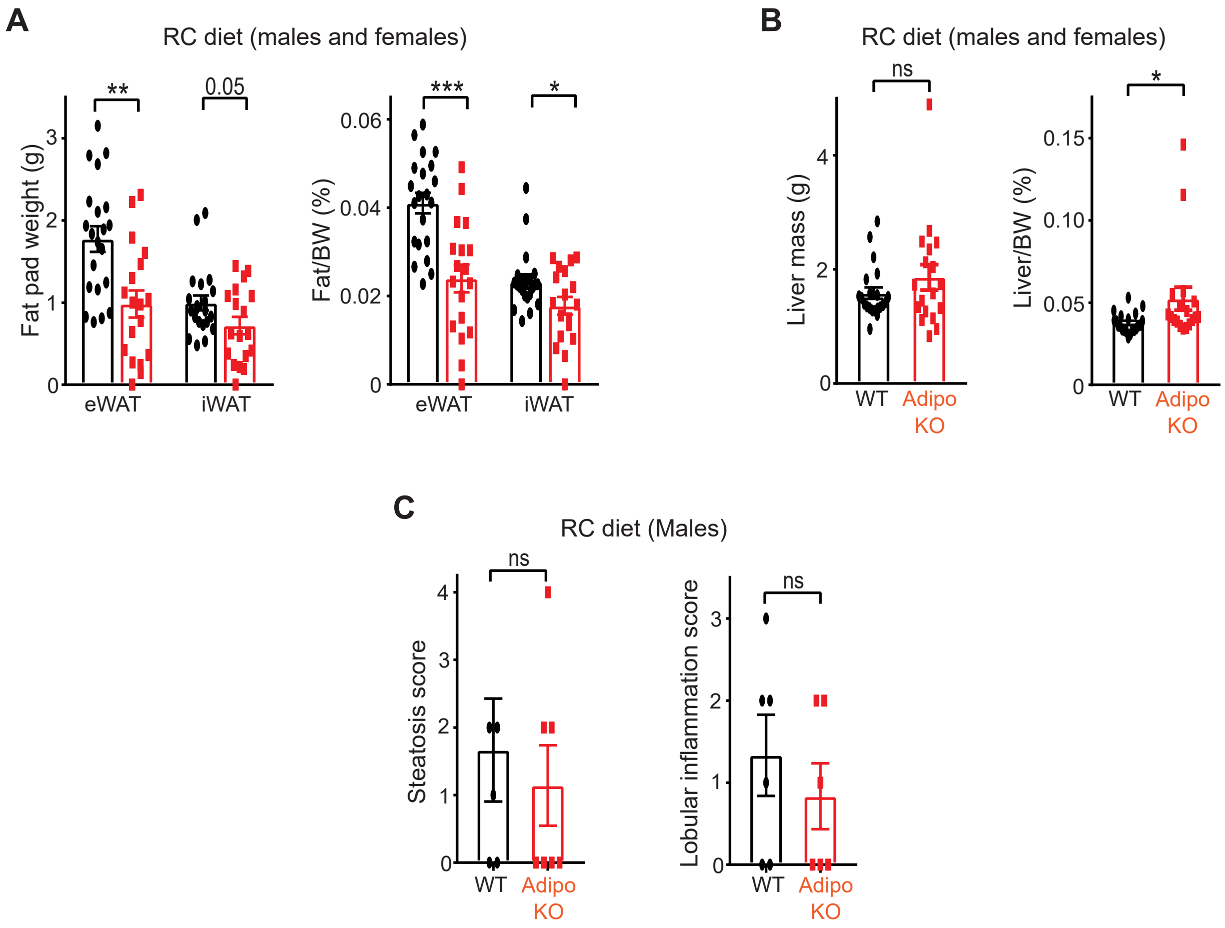

Supplementary Figure 5

Supplementary Figure 5. Tissue weights, steatosis and inflammation score of WT and Adipo KO mice in aging. (A) Total mass of epididymal (eWAT) and inguinal (iWAT) fat pads and their corresponding ratio of fat pad over body weight from WT (n=21) and Adipo KO (n=18) males and females mice upon aging under regular chow diet. **(B)** Liver mass and ratio of liver mass over body weight from WT (n=21) and Adipo KO (n=18) males and females mice upon aging under regular chow diet. **(C)** Steatosis and lobular inflammation score derived from liver sections of WT and Adipo KO (n=6-7) mice (males) upon aging under regular chow diet. Data are represented as Mean ±SEM. Two-tailed unpaired t-test was used in **A-C** where *, ** and *** represents $p<0.05$, $p<0.01$ and $p<0.001$ respectively and 'ns' denotes no significance.

Full unedited gels for Figure. 2j

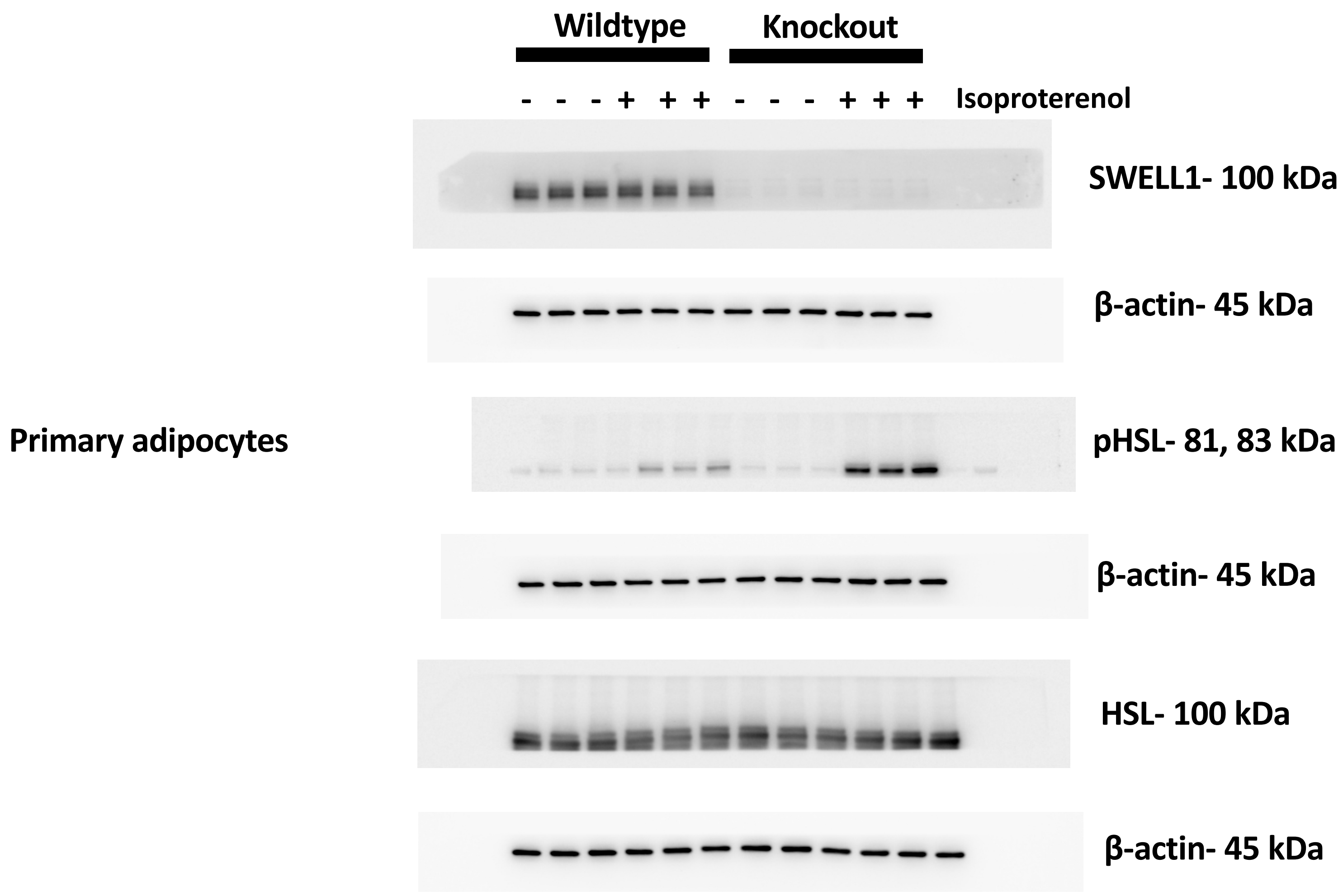

Full unedited gels for Figure. 5i

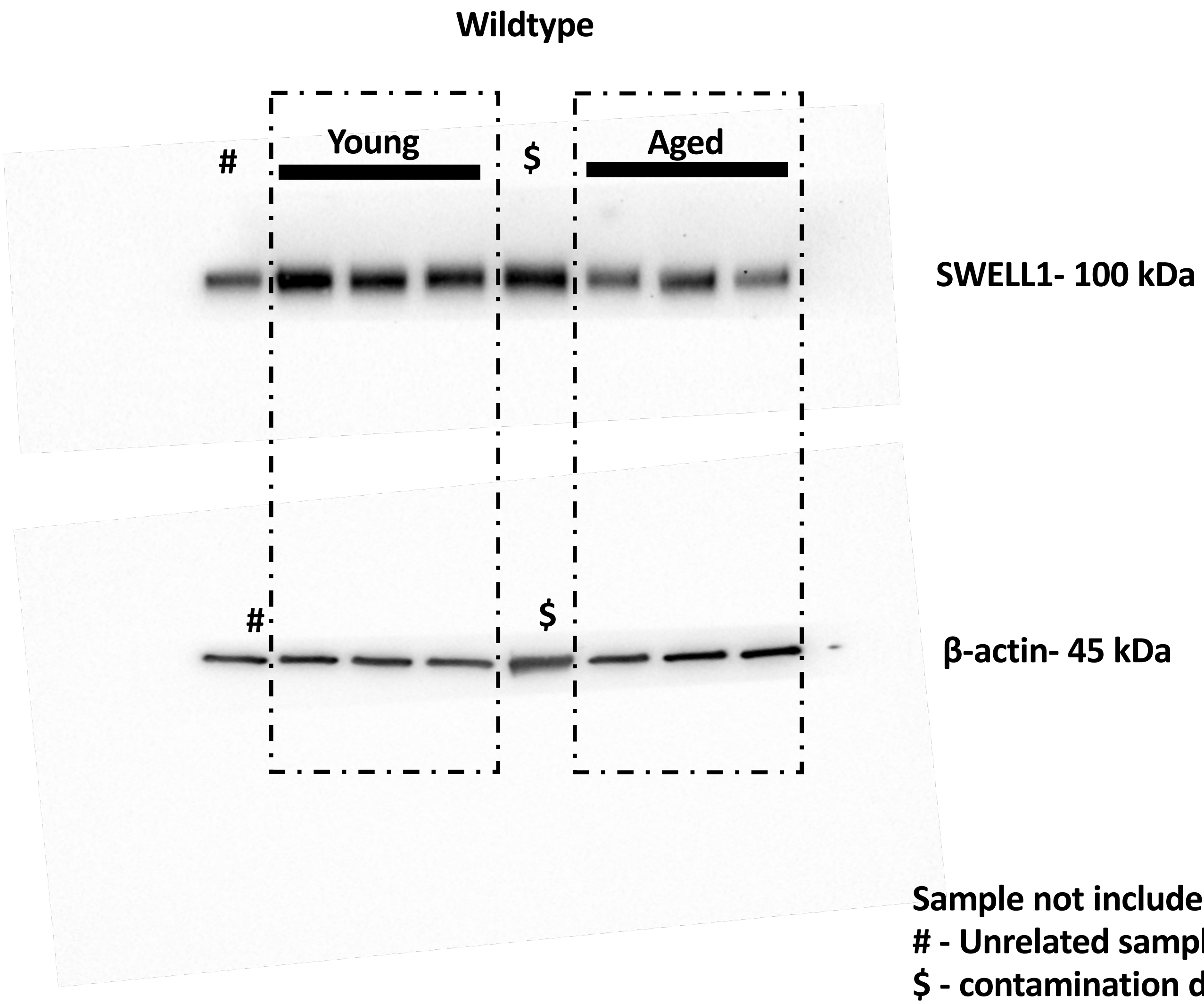

Sample not included in the analysis:
# - Unrelated sample
$ - contamination due to blood
